# Sleep Fragmentation as a Diagnostic Biomarker of Traumatic Brain Injury

**DOI:** 10.1089/neur.2025.0050

**Published:** 2025-06-09

**Authors:** Grant S. Mannino, Christian R. Baumann, Mark R. Opp, Rachel K. Rowe

**Affiliations:** ^1^ Department of Integrative Physiology, University of Colorado Boulder, Boulder, Colorado, USA.; ^2^ Department of Neurology, University Hospital of Zurich, Zurich, Switzerland.

**Keywords:** biomarker, concussion, mouse, sleep fragmentation, state transitions

## Abstract

Sleep disturbances are among the most prevalent and persistent consequences of traumatic brain injury (TBI), yet they remain underutilized as clinical indicators of injury status. In this perspective, we propose that sleep fragmentation—defined as the frequency of transitions between sleep and wakefulness—represents a functional, scalable, and underrecognized diagnostic biomarker of TBI. Drawing on empirical findings from a mouse model of diffuse TBI, we show that summary measures of sleep fragmentation and duration can reliably distinguish injured from uninjured animals using dimensionality reduction and machine learning techniques. Current biomarkers such as glial fibrillary acidic protein and neurofilament light chain provide valuable insights into structural damage but offer limited information about how injury affects behavior and day-to-day function. Sleep-based metrics, by contrast, reflect neural network integrity and capture ongoing physiological disruption. Critically, these metrics can be collected non-invasively, longitudinally, and in real-world settings using actigraphy, making them a practical complement to blood-based diagnostics that require biological sampling and specialized laboratory infrastructure. Our analysis demonstrates that sleep metrics collected over 48 h post-injury—specifically the number of sleep–wake transitions—carry a strong diagnostic signal. Sleep metrics offer a behaviorally grounded complement aligned with the goals of precision medicine and functional assessment. With further validation, these features may also support monitoring recovery or stratifying injury severity. This perspective highlights sleep fragmentation as a non-invasive diagnostic biomarker for TBI with the potential to enhance individualized monitoring and support early detection efforts in both research and clinical settings.

## Bridging the Biomarker Gap with Functional Sleep Metrics

Traumatic brain injury (TBI) remains a significant public health challenge, in part due to the heterogeneity of its clinical presentation and recovery trajectories.^1–4^ Despite major advances in neuroimaging and blood-based biomarkers, the field of TBI continues to lack functional, scalable tools for diagnosing injury subtypes and tracking recovery. Mild TBI (mTBI), or concussion, accounts for much of this complex global health issue, encompassing up to 80% of all TBIs.^5^ While many mTBI patients fully recover, a significant subset experiences persistent cognitive, emotional, and physical symptoms.^6^ Current biomarkers such as glial fibrillary acidic protein (GFAP), neurofilament light chain (NfL), and S100B reflect neural damage but offer limited insight into how injury affects behavior and quality of life.^7^ Moreover, their collection often requires specialized laboratory infrastructure and invasive sampling, posing challenges for repeated, real-time monitoring across diverse care settings. Biomarkers are often classified by their clinical utility: diagnostic (to detect the presence of injury), prognostic (to predict long-term outcomes), monitoring (to track disease progression), or predictive (to anticipate treatment response). In the context of TBI, the field remains in need of accessible tools that can support diagnostic accuracy while also laying the groundwork for future prognostic and monitoring applications.

Sleep disturbances are among the most common and persistent consequences of TBI, affecting more than half of all survivors—a significantly higher prevalence than in the general population.^8–12^ While often labeled a secondary symptom, these disturbances may reflect primary disruption in neural network function and thus offer a window into injury severity. Given the neural networks involved in sleep regulation, sleep behavior may serve as a sensitive and non-invasive indicator of brain dysfunction. Sleep fragmentation, defined as the frequency of transitions between sleep and wake states, may be a behavioral signature of injury-related network instability.

Often, post-injury sleep disturbances manifest as an acute increase in sleep with marked disruptions to sleep continuity, that is, fragmentation.^13–15^ This post-injury phenotype likely reflects underlying injury mechanisms that impede typical sleep behavior.^13,14,16^ In this perspective, we focus on the diagnostic utility of sleep fragmentation as a functional biomarker of TBI. Drawing on empirical evidence from a mouse model, we demonstrate that simple, summary sleep features can accurately distinguish injured from uninjured animals using machine learning classification. We propose that measures of sleep fragmentation offer a functional, non-invasive, and scalable tool that complements existing biological biomarkers and fills a critical gap in the assessment of injury status.

Importantly, our goal is to advance a diagnostic behavioral marker that may be used in parallel with blood-based or imaging-based measures to enhance early diagnosis. Recent findings from our group further support the prognostic utility of sleep fragmentation: In a separate analysis, we showed that sleep–wake transition metrics worsen progressively with increasing TBI severity.^17^ This pattern suggests that fragmentation not only differentiates injury from control but also provides a graded signal reflective of injury burden. Such findings position sleep fragmentation as a candidate prognostic indicator that could help stratify patients by injury severity and anticipated recovery needs.

## The Role of the “Behavioral Biomarker”

Integrating behavioral biomarkers—such as sleep–wake transition metrics—with established biological indicators offers a more complete and functional assessment of TBI severity and recovery.^18,19^ Sleep fragmentation, or disrupted sleep continuity, is a well-documented consequence of TBI^20–22^ that is associated with the development of long-term neurological complications.^23–25^ While blood- and cerebrospinal fluid-based biomarkers such as GFAP and S100B provide objective measures of neural damage, they often fail to capture the behavioral and quality-of-life impairments that shape day-to-day functioning. Behavioral biomarkers, such as sleep metrics, are especially valuable for capturing real-time functional consequences of injury.

In this context, the current utility of sleep fragmentation is primarily diagnostic, identifying TBI-related disruption of normal sleep–wake architecture. By combining molecular and behavioral biomarkers, clinicians can improve their ability to identify a brain injury, even in the absence of structural lesions, and tailor individualized treatment strategies. Over time, such biomarkers may also gain prognostic value if longitudinal patterns of sleep disruption are shown to correlate with recovery trajectories. To achieve this, future studies should evaluate sleep fragmentation in diverse injury cohorts over extended time periods, assessing whether acute-phase fragmentation predicts long-term outcomes such as cognitive impairment, emotional dysregulation, or return to function. Incorporating sleep data into prospective clinical studies alongside traditional biomarkers and outcome measures would be a critical step toward validating sleep fragmentation as a prognostic tool.

Incorporating sleep metrics into clinical workflows also enhances the predictive value of biological measures. Although some biomarkers remain elevated for weeks or months after injury, their persistence does not always align with ongoing symptoms.^26^ In contrast, sleep disturbances strongly correlate with patient-reported outcomes and quality of life, indicating they are a sensitive marker for functional impairment and recovery status.^27,28^ Disrupted sleep is also linked to elevated levels of neurodegeneration-related biomarkers, suggesting that poor sleep quality signals more severe or ongoing pathology.^29–31^ A combined biomarker approach leveraging both biological and behavioral measures can support a more patient-centered model of concussion management.

A major strength of indices of sleep fragmentation as a supplemental TBI biomarker lies in their accessibility and potential for continuous, real-world monitoring. Unlike biomarkers that require invasive procedures to obtain samples at isolated time points, sleep metrics can be collected non-invasively and longitudinally through wearable devices or home-based monitoring systems.^32^ While electroencephalogram (EEG) provides high-resolution sleep staging, it is costly and difficult to implement outside of research or inpatient clinical settings. In contrast, actigraphy-based wearables offer scalable alternatives that align more closely with outpatient and real-world monitoring needs. This enables clinicians to assess neural function dynamically, detect evolving symptoms, and adjust interventions in real time. Thus, sleep fragmentation offers not only a diagnostic readout of injury but also the infrastructure for future monitoring or prognostic utility. Importantly, because sleep disturbances contribute directly to cognitive and emotional challenges following TBI, tracking these patterns provides a layer of clinically meaningful insight that complements physiological assessments and enhances the personalization of care.^33^

## Empirical Support: Experimental Approach

To provide empirical support for the use of sleep fragmentation as a functional biomarker of TBI, we employed a well-characterized preclinical mouse model. Adult male and female C57BL/6J mice (*n* = 97) were randomly assigned to receive either a midline fluid percussion injury (TBI group) or sham surgery. All procedures were approved by the Institutional Animal Care and Use Committee at the University of Colorado Boulder and conducted in accordance with NIH guidelines.

Mice were housed individually under a 12-h light:dark cycle in a temperature-controlled environment and provided with food and water *ad libitum*. Physiological parameters were collected to determine sleep–wake behavior using a non-invasive piezoelectric monitoring system throughout a 48-h period following brain injury.^13,34–36^ Mice were acclimated to the cages for at least 5 days prior to data collection.

The midline fluid percussion injury was delivered under isoflurane anesthesia via a 3-mm craniectomy centered on the sagittal suture.^13,37,38^ Sham animals underwent the same surgical preparation without the injury impact. All injuries were administered during the light period (zeitgeber time 4–6), with non-invasive recordings starting immediately afterward.

Sleep fragmentation, as evidenced by the number of sleep–wake transitions, and total sleep time were determined in 1-h segments. A transition was defined as any shift between sleep and wake states, determined from the piezoelectric signal decision statistics analyzed at 2-s intervals.^17^ Hourly values were used to generate summary features for each mouse: the mean, standard deviation (SD), and range of sleep–wake transitions and sleep duration (minutes slept).

For downstream analyses, these six summary features were used to characterize sleep behavior across the 48-h post-injury window. To assess whether time of day influences diagnostic sensitivity, we further disaggregated sleep metrics into the light and dark periods, corresponding to the mouse’s rest and active phases, respectively. This allowed us to evaluate whether injury-related changes in sleep fragmentation were more detectable during the biologically preferred sleep phase.

All data preprocessing, dimensionality reduction, and classification analyses were conducted in R, using standardized and reproducible workflows. Detailed methods and statistical procedures are provided in the Supplementary Data.

## Functional Sleep Metrics Differentiate TBI from Sham Animals Across Diurnal Periods

**FIG. 1.**
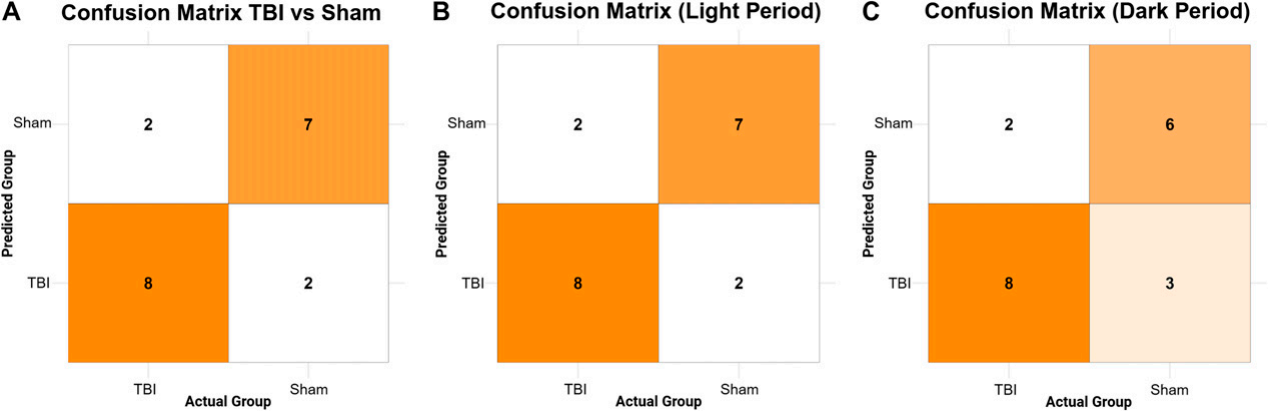
Sleep-based features accurately classify TBI versus sham using random forest models across light and dark periods. Post-injury sleep behavior differentiates TBI from sham animals with high accuracy using a random forest classifier trained on 48-h summary metrics of sleep fragmentation and sleep duration. **(A)** The model, trained on 80% of the dataset and tested on the remaining 20% (*n* = 19), used six *z*-scored features—mean, standard deviation, and range of both transitions and minutes slept per hour—and correctly classified 8 of 10 TBI and 7 of 9 sham animals. **(B)** A classifier trained and tested using light-period data also correctly classified 8 of 10 TBI and 7 of 9 sham animals. **(C)** A model using dark-period data correctly classified 8 of 10 TBI and 6 of 9 sham animals. In all panels, the intensity of orange shading reflects the number of animals correctly or incorrectly classified, with darker shades indicating a higher number of predictions in that cell. TBI, traumatic brain injury.

Analysis of post-injury sleep behavior revealed clear group-level differences between TBI and sham animals based on the number of sleep–wake transitions and total sleep duration. A random forest classifier trained on six summary sleep features (mean, SD, and range of both transitions and minutes slept per hour) demonstrated strong performance using the combined 48-h dataset. The classifier achieved an overall accuracy of 78.9% (95% confidence interval [CI]: 54.4–93.9%), with a balanced accuracy of 78.9%, sensitivity of 80.0%, and specificity of 77.8%. Cohen’s κ was 0.58, indicating moderate to substantial agreement between predicted and actual classifications. The classifier significantly outperformed the no-information rate (*p* = 0.017), highlighting its reliability in distinguishing injury condition (Fig. 1A).

To evaluate the impact of diurnal phase, we next trained separate models using only light-period or dark-period data. During the light period, the classifier achieved the highest performance overall, with 78.9% accuracy (95% CI: [54.4–93.9%]), and a balanced accuracy of 78.9%, sensitivity of 77.8%, and specificity of 80.0%. Cohen’s κ was 0.58, indicating moderate agreement between predicted and actual classifications. During the dark period, classification remained above chance with 73.7% accuracy (95% CI: [48.8–90.9%]), with a balanced accuracy of 73.7%, but with reduced sensitivity (66.7%) and greater misclassification of sham animals (Fig. 1B, C). Specificity reached 80% and Cohen’s κ was 0.47, indicating moderate agreement between predicted and actual classifications.

**FIG. 2.**
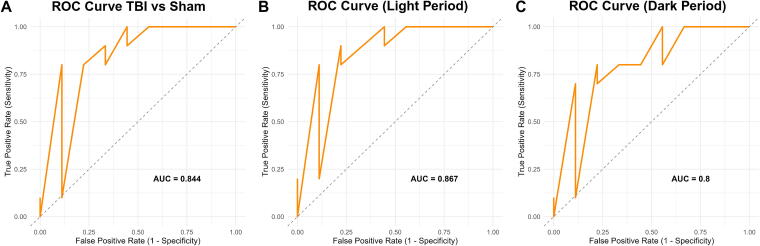
Receiver operating characteristic (ROC) analysis confirms strong classification performance of sleep-based features across light and dark periods. ROC curves illustrate the performance of random forest classifiers trained to distinguish TBI from sham animals based on post-injury sleep behavior. **(A)** The model using 48-h summary features achieved an area under the curve (AUC) of 0.844, confirming strong discriminative ability. **(B)** A model restricted to sleep data from the light period yielded an AUC of 0.867, reflecting excellent classification performance when mice are normally asleep. **(C)** The model using data from the dark period also performed well, with an AUC of 0.800, indicating that sleep disturbances during the active phase retain diagnostic value, though with slightly reduced sensitivity–specificity balance. Higher AUC values indicate better model performance across classification thresholds. Results support the utility of both rest- and active-phase sleep metrics for identifying TBI status based on non-invasive measures of sleep fragmentation and sleep loss. A dip in the ROC curve near the midrange threshold likely reflects instability due to the limited test set size and thresholding artifacts common in small-sample classification models. TBI, traumatic brain injury.

Receiver operating characteristic analysis confirmed strong model performance across all periods. The 48-h combined analysis yielded an area under the curve (AUC) of 0.844 (Fig. 2A), while the light-period model demonstrated an AUC of 0.867 (Fig. 2B). The dark-period model yielded an AUC of 0.800 (Fig. 2C), indicating that diurnally segmented sleep data may enhance diagnostic signal.

**FIG. 3.**
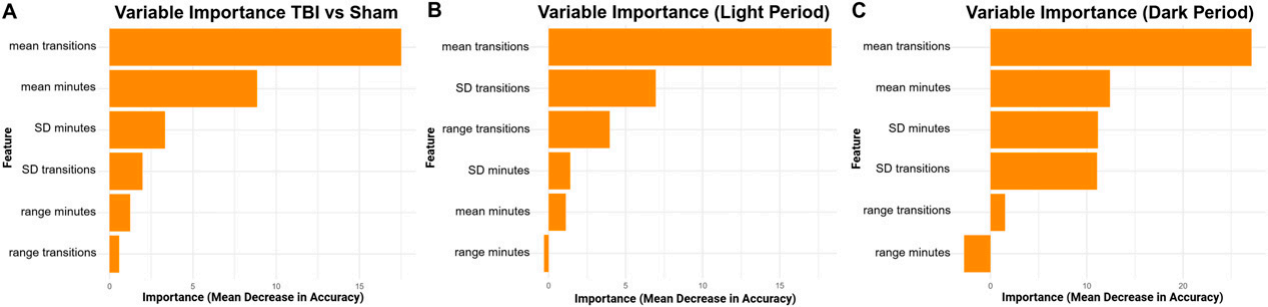
Sleep fragmentation is the strongest predictor of TBI classification performance. Variable importance analysis from the random forest models revealed that the mean number of sleep–wake transitions per hour was the most influential feature for distinguishing TBI from sham animals. **(A)** While transitions were the top predictor overall, **(B)** light-period models, which capture the normal sleep phase, showed that all three transition metrics were the most informative. **(C)** In contrast, during the dark period, when mice are typically awake, the model also relied on mean minutes slept and standard deviation of minutes slept, suggesting that abnormal sleep quantity during the active phase also contributed to classification. TBI, traumatic brain injury.

Variable importance analysis revealed that the mean number of sleep–wake transitions per hour consistently ranked as the top predictive feature. For the 48-h combined analysis, the mean number of sleep–wake transitions per hour and the mean minutes slept per hour were the most influential features contributing to classification accuracy. Additional contributions came from the variability and range of both metrics, suggesting that consistency of sleep behavior also plays a role in distinguishing injury status (Fig. 3A). The light-period model revealed that the mean number of transitions per hour was the most influential feature, followed by the SD and range of transitions. Sleep duration features contributed less, with variability and range of minutes slept showing minimal predictive power (Fig. 3B). The dark-period model showed that the mean number of sleep–wake transitions was the strongest contributor to model accuracy, followed by both the mean and SD of minutes slept. Variability in transitions also contributed, while range of sleep duration played a minimal role in classification (Fig. 3C).

**FIG. 4.**
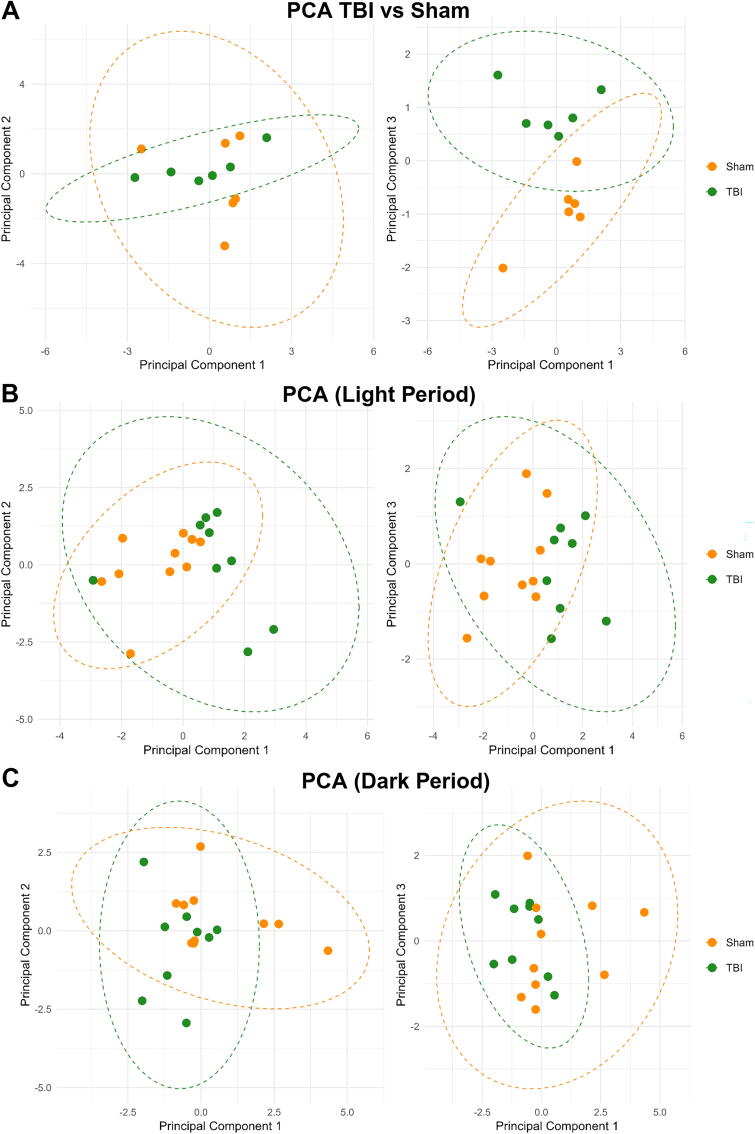
Principal component analysis (PCA) captures distinct sleep–wake disturbances after TBI across light and dark periods. PCA of post-injury sleep behavior reveals separation between TBI and sham animals. Each analysis used six *z*-scored summary features over 48 h post-injury. **(A)** PCA of 48-h sleep behavior shows clear separation between TBI and sham groups along PC1, which captures the largest share of variance and is driven primarily by sleep–wake transition metrics. TBI animals exhibit higher and more variable PC1 scores than sham controls. **(B)** PCA of light-period data also demonstrated group separation along PC1. PC2 did not differ between groups, supporting the interpretation that sleep fragmentation during the rest phase contributes strongly to group-level differences. **(C)** PCA of dark-period data similarly revealed significant separation along PC1, while PC2 showed no statistically significant difference. PC, principal component; TBI, traumatic brain injury.

Principal component analysis (PCA) supported the classification findings. In the combined dataset, the first principal component (PC1) accounted for a substantial portion of variance (41.5%) and was primarily driven by negative loadings on transitions (mean, SD, and range) and a positive loading from sleep duration variability. A one-way analysis of variance on PC1 revealed a significant group effect, *F*(1, 95) = 46.69, *p* < 0.0001; η^2^ = 0.33, 95% CI: [0.21–1.00]. Post-hoc tests confirmed significantly higher PC1 scores in TBI animals (difference = 1.80, 95% CI: [1.28–2.33], *p* < 0.001) (Fig. 4A).

When analyzed separately, PC1 also captured meaningful variance during both light (*p* = 0.017, η^2^ = 0.29, 95% CI: [0.04–1.00]) and dark periods (*p* = 0.046, η^2^ = 0.21, 95% CI: [0.00–1.00]), with group separation strongest during the light cycle (Fig. 4B, C). The second principal component (PC2) showed less separation between groups in both cases (light period: *p* = 0.956, η^2^ < 0.001; dark period: *p* = 0.164, η^2^ = 0.11). Principal component loadings for the 48-h light and dark period PCA analyses are provided in Supplementary Table S1. PC1 consistently captured variance in sleep–wake transitions, while the PC2 and the third principal component (PC3) reflected additional contributions from variability in sleep duration.

Together, these analyses demonstrate that acute summaries of sleep behavior—particularly sleep fragmentation—can accurately classify TBI and sham animals, especially when aligned with the species’ primary sleep phase. These findings support the utility of sleep fragmentation as a sensitive and scalable biomarker of post-injury neurophysiological disruption. A full comparison of classifier performance across the combined, light, and dark datasets is provided in Supplementary Table S2.

## Toward Functional, Scalable Biomarkers for TBI

Our findings demonstrate that a 48-h assessment of sleep behavior can effectively distinguish between control, uninjured mice, and mice subjected to TBI, highlighting the diagnostic value of sleep–wake transitions and total sleep duration. The strong performance of the random forest classifier and the robustness of the PCA provide compelling evidence that sleep fragmentation is a consistent and quantifiable feature of TBI-related neurophysiological disruption.

PCA revealed that the PC1 captured meaningful variance in sleep behavior, largely driven by a reduction in sleep–wake transition consistency and increased variability in total sleep time—both characteristics predominantly observed in injured animals. Exploratory analysis of PC1 and PC3 offered additional evidence of group separation, suggesting that multiple dimensions of sleep behavior contribute to the observed phenotype. Our variable importance analysis confirmed that mean transitions per hour was the strongest predictor of injury overall, particularly during the light period, when mice are normally asleep and consolidated sleep is expected. In this context, fragmented sleep emerged as a clear indicator of injury status.

By contrast, during the dark period, when control mice are mostly active, mean minutes slept became one of the top predictors. This pattern suggests that excessive sleep during the active phase (analogous to excessive daytime sleepiness in humans) may also signal dysfunction following TBI. These findings highlight the value of diurnal segmentation in enhancing sensitivity to injury-related changes in sleep behavior. Clinically, this suggests that the diagnostic utility of sleep metrics may depend on when sleep is measured. Because humans sleep predominantly during the dark period (unlike nocturnal rodents), these results support targeting structured, habitual rest periods—such as overnight sleep—for optimal diagnostic signal. Segmenting behavioral sleep data by diurnal period may enhance both sensitivity and specificity when developing functional biomarkers for TBI.

A key strength of this approach is its simplicity and scalability. The use of 48-h summary sleep metrics enables researchers and clinicians to avoid complex, resource-intensive analyses while retaining diagnostic value. This efficiency aligns well with real-world constraints in both research and clinical settings. With the growing availability of wearable technologies and passive monitoring systems, continuous sleep assessment is not only feasible but also increasingly practical for early detection, progress tracking, and individualized treatment planning in TBI care.^32,39,40^

There are limitations that should be acknowledged. In this study, a small subset of TBI mice were misclassified, likely due to sample size constraints and variability within the injury group. Misclassification of these mice highlights the importance of expanding cohorts and incorporating advanced validation techniques such as bootstrapping or cross-validation to enhance generalizability. Additionally, further work is needed to determine the mechanistic pathways that underlie altered sleep–wake behavior following TBI, particularly in relation to inflammation, neural network connectivity, and neurodegenerative cascades.

From a translational standpoint, this study presents a robust, interpretable framework for integrating behavioral sleep metrics into the neurotrauma toolkit. The alignment of high model performance with biologically grounded predictors supports the feasibility of this approach. Importantly, it bridges preclinical findings with future clinical application, offering a scalable, non-invasive strategy for characterizing TBI severity and heterogeneity. By advancing sleep fragmentation as a functional biomarker, we take a critical step toward precision medicine in brain injury diagnosis and care.

## Abbreviations Used

ANOVA: Analysis of Variance
CI: Confidence Interval
CSF: Cerebrospinal Fluid
EEG: Electroencephalogram
GFAP: Glial Fibrillary Acidic Protein
mTBI: Mild Traumatic Brain Injury
NfL: Neurofilament Light Chain
η²: Eta Squared (effect size)
PC1, PC2, PC3: Principal Components 1, 2, 3
PCA: Principal Component Analysis
ROC: Receiver Operating Characteristic
TBI: Traumatic Brain Injury
