# Supplementary material for: Sleep Fragmentation as a Diagnostic Biomarker of Traumatic Brain Injury
Source: Neurotrauma Rep. 2025 Jun 9;6(1):482–90. doi: 10.1089/neur.2025.0050 (PMC12167842; doi:10.1089/neur.2025.0050)
Supplement: Supplementary Data [file neur.2025.0050_supplementary_data.docx]

**Supplementary Material**

Animals

All animal studies were approved by the Institutional Animal Care and Use Committee (IACUC) at the University of Colorado Boulder (protocol 2819) and conducted in accordance with the National Institutes of Health (NIH) guidelines for the care and use of laboratory animals. We evaluated sleep in wild-type (C57BL/6J) male and female mice (*n* = 97) using the non-invasive piezoelectric cage system. All mice were bred in-house from breeder pairs obtained from Jackson Laboratories (Bar Harbor, ME). For the study, mice were singly housed and maintained on a 12-hour light:dark cycle (200 lux, cool white, fluorescent light) at an ambient temperature of 24°C ± 2°C. All mice were acclimated to non-invasive piezoelectric sleep cages for a minimum of 5 days prior to initiation of data collection. Mice were fed a normal diet of standard rodent chow, and food and water were available *ad libitum*.

Midline fluid percussion injury

Mice were anesthetized with 5% isoflurane in 100% oxygen for 5 minutes, then secured in a stereotaxic frame with a continuous flow of 2.5% isoflurane via a nosecone. Eye ointment was applied, and the surgical site was cleaned with alternating betadine and ethanol. A midline scalp incision was made to expose the skull, and a 3 mm craniectomy was trephined along the sagittal suture, midway between bregma and lambda. An injury hub, constructed from a Luer-Loc needle hub, was affixed over the craniectomy using cyanoacrylate gel and methyl methacrylate (Hygenic Corp., Akron, OH). The hub was filled with sterile saline and sealed with a modified syringe tip to prevent debris and air exposure.

Twenty-four hours later, mice were re-anesthetized with 5% isoflurane in 100% oxygen for 3 minutes. The cap was removed, and the dura was inspected to confirm its integrity. The hub was then refilled with saline and connected via extension tubing to a fluid percussion injury device (Custom Design and Fabrication, Virginia Commonwealth University, Richmond, VA). Once a toe-pinch reflex was observed, the pendulum was released, delivering a fluid pulse to the intact dura, consistent with our published protocols (1-4).

Sham animals underwent the same procedures, including hub placement and device attachment, but did not receive the fluid pulse. After injury or sham procedure, mice were briefly re-anesthetized, the injury site was cleaned, and the scalp was closed with sutures. Bacitracin was applied, and animals were placed in a heated recovery cage. All injuries were performed between zeitgeber time (ZT) 4 and 6.

Sleep Data Collection

Sleep-wake behavior was recorded for 48 hours post-injury using a non-invasive piezoelectric system (Signal Solutions, Lexington, KY, USA) as previously described (1, 5-7). Sleep-wake transitions were identified based on decision statistics calculated every 2 seconds, with transitions classified as sleep if the decision statistic exceeded a predefined threshold. Transitions from wake to sleep and vice versa were summed, providing the total number of sleep-wake transitions. Total minutes slept and sleep-wake transitions were quantified on an hourly basis (*i.e.,* hour segments). Determination of sleep-wake behavior based on physiological parameters was done by investigators blind to experimental conditions. The system recorded the total number of minutes spent asleep and the total number of sleep-wake transitions for each mouse, with data collected every 60 minutes across the entire 48-hour observation period. These data were used to calculate several summary measures for each mouse: the mean, standard deviation, and range of minutes slept per hour and the mean, standard deviation, and range of sleep-wake transitions per hour.

Data Analysis in R

All statistical analyses were performed in R version 4.0.3 (R Foundation for Statistical Computing, Vienna, Austria). The data were first cleaned and prepared by excluding any incomplete data points, ensuring that only valid and consistent measures were included in the analysis. The primary analyses were performed using the tidyverse package for data manipulation and visualization.

*Principal Component Analysis (PCA)*

Principal component analysis (PCA) was performed on the summary features (mean, standard deviation, and range of total minutes slept and sleep-wake transitions) to reduce the dimensionality of the data and identify the underlying components that best differentiate the Sham and TBI groups. PCA was carried out using the prcomp() function from the base stats package in R, with data centered and scaled to account for differences in variable units.

The first two principal components (PC1 and PC2) were used in the primary analysis, but an additional exploratory analysis was conducted using PC1 vs. PC3 to assess the potential for separation between the groups along the third principal component. This was intended to further explore whether sleep features, as captured by the first three principal components, provide meaningful group separation beyond the first two components.

*Random Forest Classification*

To assess the ability of sleep features to classify Sham vs. TBI mice, we trained a random forest classifier using the randomForest package in R. The dataset was randomly split into 80% training and 20% testing subsets. The classifier was trained using the six sleep-related features (mean, standard deviation, and range of minutes slept and sleep-wake transitions) for each animal, with 500 trees and default parameters. Model performance was evaluated on the test set using accuracy, sensitivity, specificity, and the area under the receiver operating characteristic (ROC) curve (AUC), which was calculated using the pROC package.

*Statistical Analysis*

The significance of differences in sleep features and PCA components between the Sham and TBI groups was evaluated using one-way analysis of variance (ANOVA). The ANOVA was performed on PC1 and PC2 to assess the group separation along these components. Post-hoc pairwise comparisons were conducted using Tukey’s HSD test for significant ANOVA results. The effect size for significant results was computed using partial eta squared (η²), which was calculated using the effectsize package in R.

In addition to the main PCA analysis, a post-hoc ANOVA was performed on PC1 and PC2, and the results were interpreted based on the calculated p-values. For exploratory purposes, PC1 vs. PC3 was plotted to investigate potential further separation between the groups.

**References**

1. Rowe RK, Striz M, Bachstetter AD, Van Eldik LJ, Donohue KD, O'Hara BF, et al. Diffuse brain injury induces acute post-traumatic sleep. PloS one. 2014;9(1):e82507.

2. Eakin K, Rowe RK, Lifshitz J. Modeling Fluid Percussion Injury: Relevance to Human Traumatic Brain Injury. In: Kobeissy FH, editor. Brain Neurotrauma: Molecular, Neuropsychological, and Rehabilitation Aspects. Frontiers in Neuroengineering. Boca Raton (FL)2015.

3. Rowe RK, Griffiths DR, Lifshitz J. Midline (Central) Fluid Percussion Model of Traumatic Brain Injury. In: Kobeissy HF, Dixon EC, Hayes LR, Mondello S, editors. Injury Models of the Central Nervous System: Methods and Protocols. New York, NY: Springer New York; 2016. p. 211-30.

4. Lifshitz J, Rowe RK, Griffiths DR, Evilsizor MN, Thomas TC, Adelson PD, et al. Clinical relevance of midline fluid percussion brain injury: Acute deficits, chronic morbidities and the utility of biomarkers. Brain Injury. 2016.

5. Saber M, Murphy SM, Cho Y, Lifshitz J, Rowe RK. Experimental diffuse brain injury and a model of Alzheimer's disease exhibit disease-specific changes in sleep and incongruous peripheral inflammation. J Neurosci Res. 2021;99(4):1136-60.

6. Rowe RK, Harrison JL, Zhang H, Bachstetter AD, Hesson DP, O'Hara BF, et al. Novel TNF receptor-1 inhibitors identified as potential therapeutic candidates for traumatic brain injury. Journal of neuroinflammation. 2018;15(1):154.

7. Rowe RK, Green TRF, Giordano KR, Ortiz JB, Murphy SM, Opp MR. Microglia Are Necessary to Regulate Sleep after an Immune Challenge. Biology (Basel). 2022;11(8).
