# Supplementary material for: Sleep Fragmentation as a Diagnostic Biomarker of Traumatic Brain Injury
Source: Neurotrauma Rep. 2025 Jun 9;6(1):482–90. doi: 10.1089/neur.2025.0050 (PMC12167842; doi:10.1089/neur.2025.0050)
Supplement: Supplementary Tables [file neur.2025.0050_supplementary_tables.docx]

## Supplementary Table 1. PCA Loadings for Sleep Summary Features Across Time Windows.

| Feature | PC1 (48h) | PC2 (48h) | PC3 (48h) | PC1 (Light) | PC2 (Light) | PC3 (Light) | PC1 (Dark) | PC2 (Dark) | PC3 (Dark) |
| --- | --- | --- | --- | --- | --- | --- | --- | --- | --- |
| Mean Minutes Slept | 0.21 | 0.43 | -0.82 | -0.18 | 0.18 | -0.94 | 0.21 | 0.41 | -0.74 |
| SD Minutes Slept | -0.4 | -0.48 | -0.17 | 0.21 | -0.63 | -0.04 | -0.28 | -0.56 | 0.02 |
| Range Minutes Slept | -0.37 | -0.44 | -0.52 | 0.22 | -0.65 | -0.24 | -0.28 | -0.45 | -0.31 |
| Mean Transitions | 0.5 | -0.1 | -0.11 | 0.49 | 0.31 | 0.11 | 0.5 | 0.08 | 0.52 |
| SD Transitions | 0.44 | -0.46 | 0.04 | 0.57 | 0.16 | -0.14 | 0.51 | -0.42 | -0.11 |
| Range Transitions | 0.47 | -0.41 | -0.12 | 0.55 | 0.12 | -0.16 | 0.53 | -0.36 | -0.25 |

Principal component loadings for six summary sleep features used in PCA analyses across the full 48-hour period, light period, and dark period. PCA was performed on z-scored feature values of sham and TBI mice (n = 97). PC1 consistently reflected sleep-wake transitions, while PC2 and PC3 captured additional variability from sleep duration measures. Loadings indicate the strength and direction of each feature’s contribution to the first three principal components. PCA: Principal Component Analysis; PC: Principal Component; SD: standard deviation

# Supplementary Table 2. Comparison of Classification Performance

| Metric | 48-Hour Combined | Light Period Only | Dark Period Only |
| --- | --- | --- | --- |
| Accuracy | **78.9%** | **78.9%** | 73.7% |
| Balanced Accuracy | **78.9%** | **78.9%** | 73.3% |
| Sensitivity | **80.0%** | 77.8% | 66.7% |
| Specificity | 77.8% | **80.0%** | **80.0%** |
| Cohen’s Kappa | **0.58** | **0.58** | 0.47 |
| AUC | 0.844 | **0.867** | 0.800 |
| PC1 η² (Effect Size) | **0.33** | 0.29 | 0.21 |
| PC1 p-value | < 0.0001 | **0.017** | 0.046 |
| PC2 η² (Effect Size) | 0.08 | **<0.001** | 0.11 |
| PC2 p-value | 0.851 | 0.956 | **0.164** |

*Note: Bolded values indicate best performance across conditions. All models trained on six z-scored summary sleep features.

Comparative analysis of sleep-derived features revealed that separating the light and dark periods continued to provide stronger classification of TBI status than using the combined 48-hour dataset. The model trained on light-period data achieved the best overall performance, with 78.9% accuracy, balanced accuracy of 78.9%, and a robust AUC of 0.867. Group separation on PC1 was statistically significant (p = 0.017) with a moderate effect size (η² = 0.29). The dark-period model also showed meaningful discriminative capacity (accuracy = 73.7%, AUC = 0.800), though PCA results indicated more modest group separation (PC1 η² = 0.21, p = 0.046). In contrast, the model trained on combined 48-hour data showed similar classification accuracy (78.9%) but lower AUC (0.844) and less specificity in group differentiation (PC1 η² = 0.33, p = 0.851). PCA: Principal Component Analysis; AUC: area under the curve.
